# Lymphatic metastasis-associated circRNA‒miRNA‒mRNA network for exploring the pathogenesis and therapeutic target of triple negative breast cancer based on whole-transcriptome sequencing analysis: an experimental verification study

**DOI:** 10.1186/s12967-022-03728-6

**Published:** 2022-11-05

**Authors:** Jiayue Luo, Dong Cao, Chuwen Hu, Zhen Liang, Yuanping Zhang, Jianguo Lai

**Affiliations:** 1 Department of Breast Surgery, Guangzhou Women and Children’s Medical Center, Guangzhou Medical University, Guangzhou, 510623 Guangdong China; 2 Department of Anesthesiology, Sun Yat-Sen Memorial Hospital, Sun Yat-Sen University, Guangzhou, 510120 Guangdong China; 3 Guangdong Provincial People’s Hospital, Guangdong Academy of Medical Sciences, 106 Zhongshan Er Road, Yuexiu District, Guangzhou, 510080 Guangdong China

**Keywords:** Whole transcriptome sequencing, Triple-negative breast cancer, Axillary lymph node metastasis, circRNA

## Abstract

**Background:**

The metastatic mechanisms of axillary lymph nodes (ALNs) in triple-negative breast cancer (TNBC) remain unclear. We aimed to identify the potential circRNA regulatory network in ALN metastasis.

**Methods:**

We performed whole transcriptome sequencing (WTS) to determine the expression profiles of RNAs and screen out differentially expressed messenger RNAs (DEMs), microRNAs (DEMis), and circRNAs (DECs) between ALN-positive and ALN-negative TNBC patients. Functional enrichment analysis and Kaplan–Meier survival analysis were utilized to unearth the potential regulatory mechanisms of the DEMs. A competing endogenous RNA (ceRNA) network was constructed using computational biology. The expression levels of DECs in cell lines were confirmed by real-time polymerase chain reaction (RT‒PCR).

**Results:**

Following WTS and differential expression analysis, 739 DEMs, 110 DEMis, and 206 DECs were identified between ALN-positive and ALN-negative TNBC patients. Functional analysis indicated that the DEMs mainly functioned in carcinogenesis and tumor progression-related pathways. ceRNA networks containing eight circRNAs, six miRNAs, and eighteen mRNAs were developed. In the ceRNA network, two mRNAs (RAB3D and EDARADD) that were significantly associated with better overall survival and one mRNA (GSR) that predicted favorable recurrence-free survival in TNBC patients were chosen for further analysis. Then, a survival-related ceRNA network containing two DECs (hsa_circ_0061260 and hsa_circ_0060876), two DEMis (hsa-miR-5000-3p and hsa-miR-4792), and three mRNAs (GSR, RAB3D, and EDARADD) was identified. Then, two candidate DECs were validated by real-time PCR.

**Conclusion:**

Our research constructed a ceRNA network that provides novel insights into the molecular mechanism of ALN metastasis and potential therapeutic targets in TNBC.

## Introduction

Triple-negative breast cancer (TNBC) accounts for approximately 15% of the total incidence of breast carcinoma diagnosed worldwide and has a relatively unfavorable prognosis [1]. For patients with TNBC, the risk of local or regional recurrence, distant metastasis, and death peaks approximately two to three years after diagnosis, and TNBC appears to be more likely to metastasize to visceral organs, such as the liver, lungs, and brain [2, 3]. In particular, metastasis of axillary lymph nodes (ALNs) in TNBC patients is a strong and independent prognostic factor that indicates a worse five-year survival rate [4]. Additionally, micrometastatic nodal involvement is associated with an increased risk of death compared with patients with negative lymph nodes [5]. However, the potential precise mechanisms of ALN metastasis remain unclear.

Noncoding RNAs (ncRNAs), mainly microRNAs (miRNAs), long noncoding RNAs (lncRNAs), and circular RNAs (circRNAs), make up most of the transcriptome and play vital roles in the oncogenesis and progression of TNBC [6]. Previous studies found that circRNAs act as sponges of the corresponding miRNAs, thus leading to the progression and chemoresistance of TNBC [7–9]. Zeng et al. discovered that circANKS1B was overexpressed in TNBC tissue and was associated with lymph node metastasis [10]. circRNAs may also act as tumor suppressors. circTADA2A-E6 was remarkably upregulated in TNBC and inhibited tumor growth and metastasis [11]. In addition, circFBXW7 sponged miR-197-3p and further inhibited TNBC progression [12]. However, there are few available studies on the functions of circRNAs in ALN metastasis in TNBC. The rapid development of next-generation sequencing technologies has made whole-transcriptome sequencing (WTS) much easier to conduct. Through WTS, we can capture both noncoding RNAs and coding RNAs in cells and tissues to determine the genetic interaction networks and the corresponding functions [13]. However, few studies have applied WTS to explore the gene expression profile in detail and to better understand the transcriptomic landscape of ALN metastasis in TNBC.

In our study, WTS was conducted on the tumor tissues from three ALN-positive and three ALN-negative patients with TNBC. Next, differential expression analysis was conducted to recognize the significant differentially expressed messenger RNAs (DEMs), miRNAs (DEMis), and circRNAs (DECs) between the ALN-positive and ALN-negative groups. Furthermore, we built a circRNA-miRNA‒mRNA network and performed functional analysis to elucidate the potential mechanisms of ALN metastasis in TNBC, which provides novel insight into the prognostic prediction and potential therapeutic application of circRNAs in the ALN metastasis of TNBC.

## Materials and methods

Three pairs of tumor tissues were acquired from six early TNBC patients (three ALN-positive and three ALN-negative patients) who underwent surgical treatment at our hospital with approval from the Ethics Committee of our hospital. Participants in this study signed informed consent forms. All samples were preserved in liquid nitrogen.

Total RNA was extracted from three ALN-positive and three ALN-negative tumor tissues using TRIzol reagent (Life Technologies, USA) according to the manufacturer’s protocol. WTS of mRNAs, miRNAs, and circRNAs was conducted by Aksomics (Guangzhou, China) using the Illumina HiSeq 3000 platform. DEMs, DEMis, and DECs were screened between ALN-positive and ALN-negative TNBC tumor tissues using the Limma package in R. A threshold of a 2-fold change and p < 0.01 was considered significantly different. The DEMs, DEMis, and DECs were visualized on heatmaps and volcano plots. The biological functions of the DEMs were further explored by functional enrichment analysis on the Kyoto Encyclopedia of Genes and Genomes (KEGG) dataset and Hallmark’s gene datasets using the cluster Profiler package in R. p values < 0.05 were considered to indicate significantly enriched pathways.

Potential miRNA‒mRNA interaction networks were explored using the miRTarBase and TargetScan databases. Then, the predicted miRNA‒mRNA regulatory relationship was integrated with the DEM data to acquire the DEMi-DEM regulatory relationship. Then, the ceRNA networks were developed using Cytoscape (version 3.6.1; http://www.cytoscape.org/).

Real-time PCR was utilized to validate the expression levels of the candidate DECs in the ceRNA networks in the following cell lines (MCF-10 A, MB231, and MB453). Specifically, total RNA was extracted from tissues with TRIzol (Life Technologies, USA), as mentioned above. Then, the PrimeScript Reagent Kit (Vazyme Biotech Co., Ltd, China) was utilized to synthesize the single-stranded cDNA. cDNA amplification was performed starting from 100 ng of total RNA. The relative expression levels of circRNAs were detected by SYBR Green Master Mix (Vazyme Biotech Co., Ltd, China) in the RT‒PCR system (Applied Biosystems Step One Plus, USA). Both divergent and convergent primers were used to validate the circular structures of circRNAs. Glyceraldehyde 3-phosphate dehydrogenase (GAPDH) was utilized as an internal control. To identify whether the circRNAs contained back-splice sites, Sanger sequencing was utilized (Beijing Genomics institution, China).

Stata/MP, version 14.0 (Stata Corp LP, College Station, TX), and R software (version 4.1.3) were employed to carry out the statistical analyses. A paired design Wilcoxon signed-rank test was conducted to accomplish the differential expression analyses in paired tumor tissue samples. Correlation analyses were carried out by Pearson’s correlation test. Quantitative real-time PCR was conducted in three replicates, and the data are expressed as the mean ± SEM. The Kaplan‒Meier method and log-rank test were utilized to compare the recurrence-free survival (RFS) and overall survival (OS) of TNBC patients based on the Kaplan‒Meier Plotter database. p < 0.05 was considered statistically significant.

## Results

**Fig. 1:**
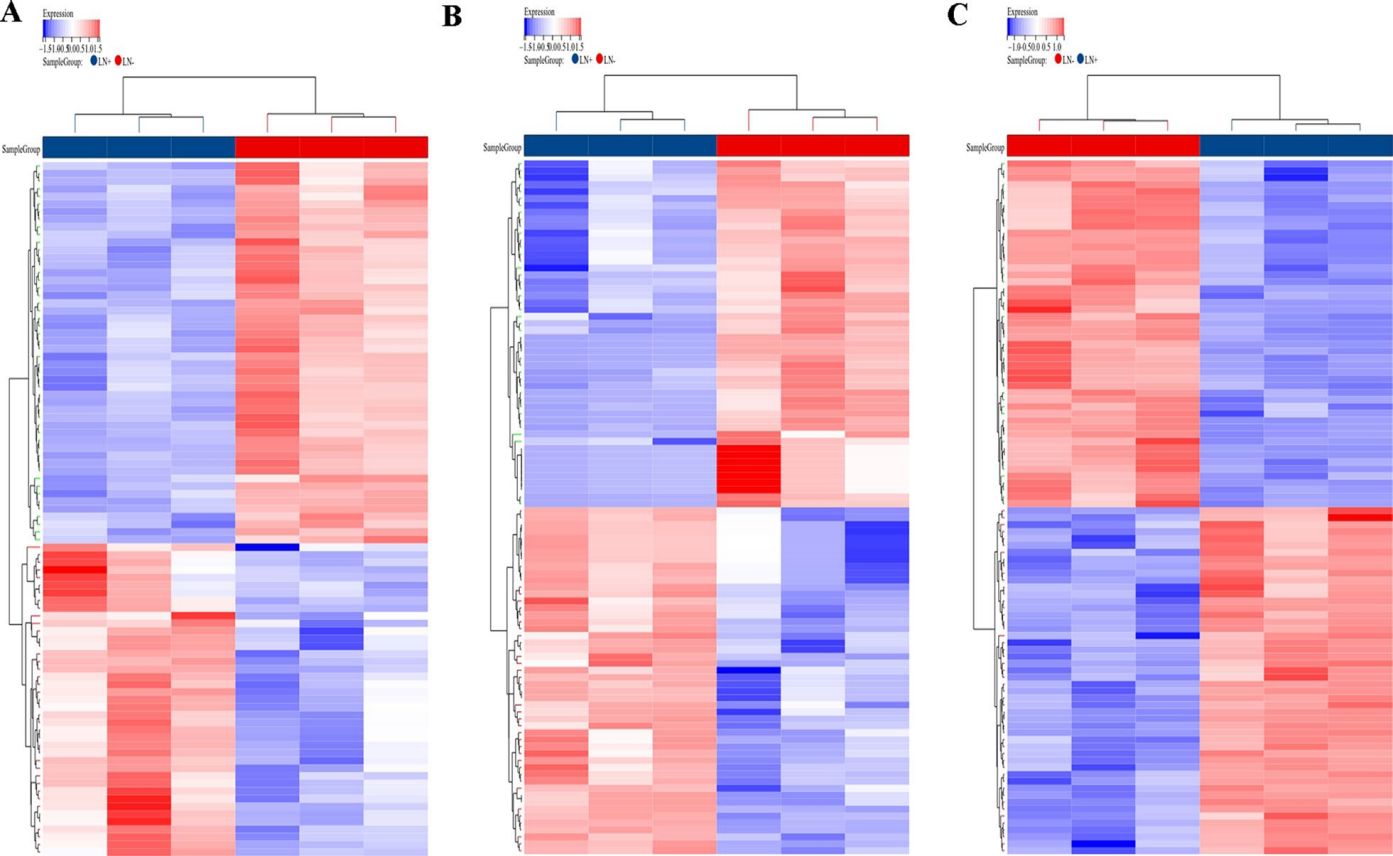
Heatmaps of differentially expressed RNAs between ALN-positive and ALN-negative TNBC patients. The differential expression of circRNAs (**A**), miRNAs (**B**), and mRNAs (**C**)

Firstly, to identify the potential ALN metastasis-associated miRNAs, circRNAs, and mRNAs, WTS was conducted on three pairs of ALN-positive and ALN-negative TNBC tissues. In total, we identified 739 mRNAs, 206 circRNAs, and 110 miRNAs that were differentially expressed, and they are presented in heatmaps (Fig. 1).

**Fig. 2:**
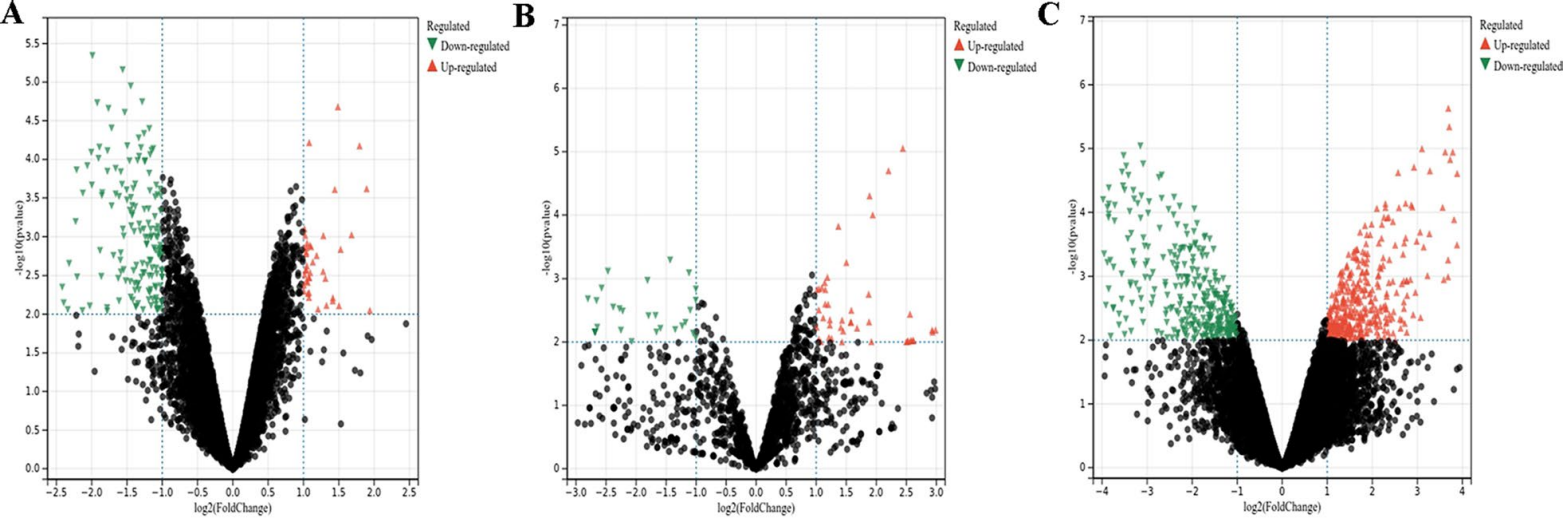
Volcano plots of differentially expressed RNAs between ALN-positive and ALN-negative TNBC patients. The differential expression of circRNAs (**A**), miRNAs (**B**), and mRNAs (**C**)

In brief, 206 DECs consisting of 41 upregulated and 165 downregulated circRNAs were identified (Fig. 2A). A total of 110 DEMis with 53 upregulated miRNAs and 57 downregulated miRNAs were identified (Fig. 2B). Meanwhile, among the 739 DEMs, 371 mRNAs were overexpressed, and 368 mRNAs were downregulated (Fig. 2C).

**Fig. 3:**
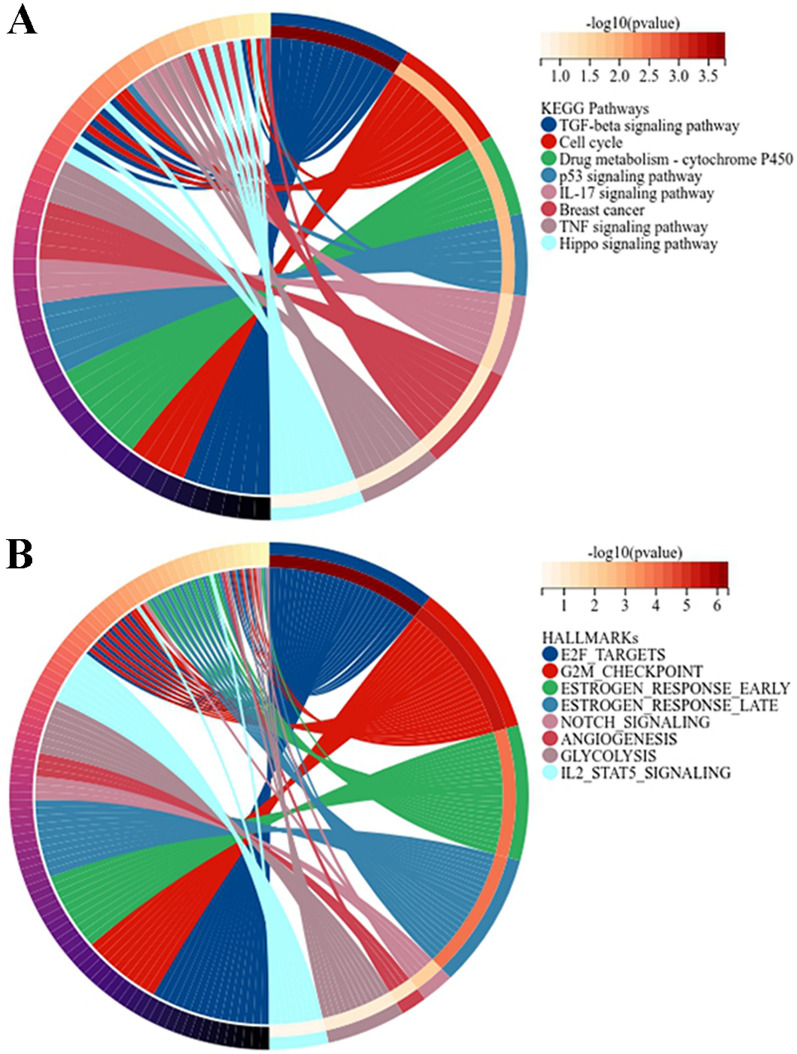
Functional enrichment analysis of DEMs. DEMs are associated with signaling pathways in KEGG (**A**) and hallmark datasets (**B**)

Then, functional enrichment analysis of DEMs was conducted. KEGG pathway analysis was performed to identify the significantly enriched pathways via clusterProfiler (version 3.14.3). Specifically, DEMs were mainly enriched in the TGF-beta signaling pathway, drug metabolism-cytochrome P450 pathway, cell cycle, p53 signaling pathway, breast cancer, TNF signaling pathway, IL-17 signaling pathway, and hippo signaling pathway (Fig. 3A). In addition, hallmark gene analysis was conducted to identify meaningful pathways based on the Molecular Signatures Database using clusterProfiler. DEMs were mainly involved in E2F targets, notch signaling, angiogenesis, the G2M checkpoint, glycolysis, the early estrogen response, the late estrogen response, and IL2 STAT5 signaling (Fig. 3B). Among these pathways, the TNF signaling pathway, cell cycle, p53 signaling pathway, breast cancer, and NOTCH signaling pathway were associated with the carcinogenesis and progression of TNBC.

**Fig. 4:**
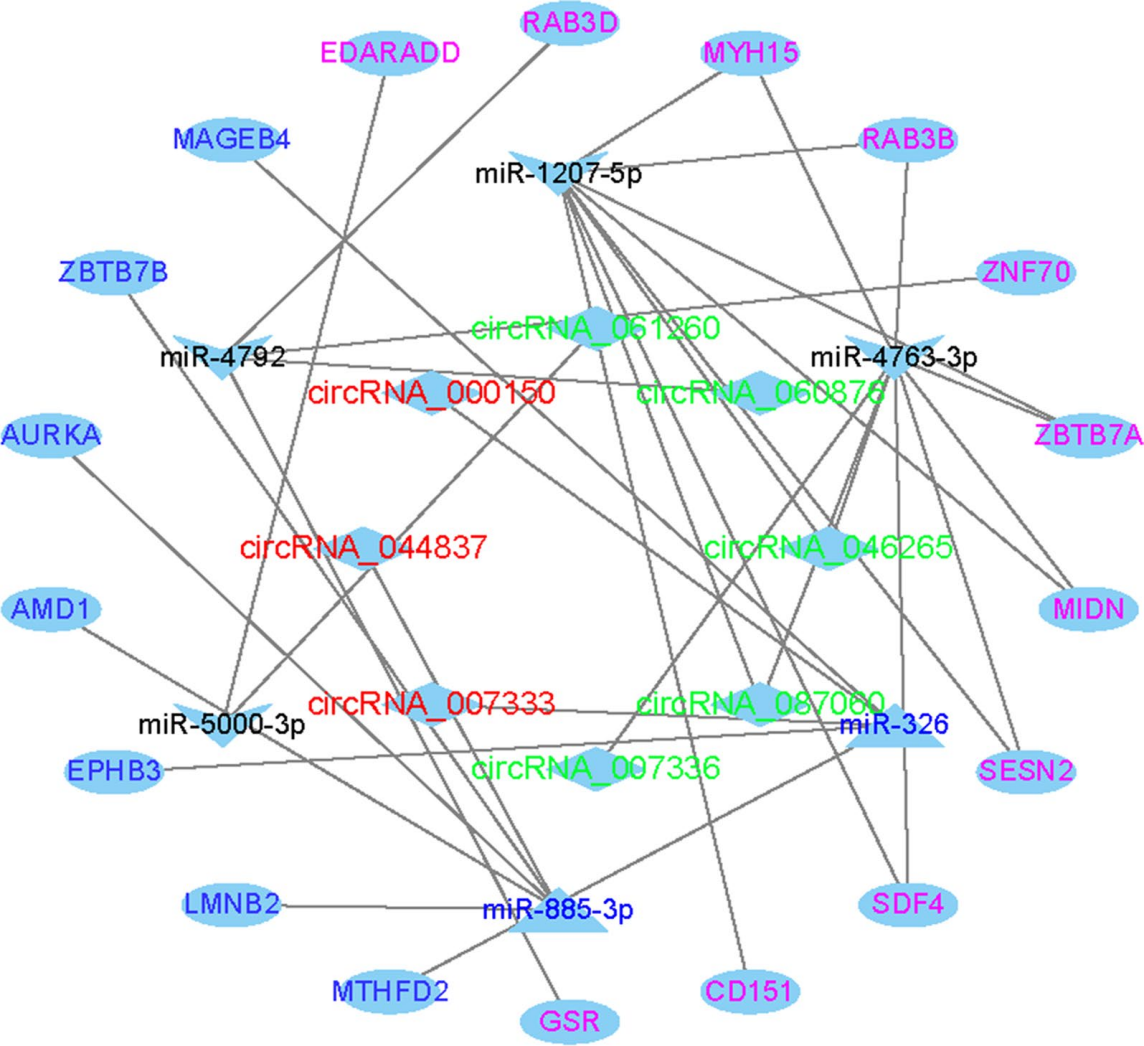
The ALN metastasis-related ceRNA network of circRNA–miRNA–mRNA in TNBC.

Next, to explore the potential vital roles of DECs and DEMis in the ceRNA network of TNBC, we constructed a circRNA-miRNA‒mRNA (ceRNA) network. First, we extracted data related to the eight top-ranked DECs (hsa_circRNA_061260, hsa_circRNA_060876, hsa_circRNA_046265, hsa_circRNA_087060, hsa_circRNA_007336, hsa_circRNA_007333, hsa_circRNA_044837, and hsa_circRNA_000150) selected from the RNA-seq data. Then, we screened the potential miRNAs targeted by the eight DECs using the circBank and CircInteractome online datasets and overlapped them with the identified DEMis. As a result, a circRNA-miRNA network was generated that consisted of 8 DECs and 6 DEMis (hsa-miR-1207-5p, hsa-miR-4763-3p, hsa-miR-326, hsa-miR-885-3p, hsa-miR-5000-3p, and hsa-miR-4792). Subsequently, miRTarBase and TargetScan were utilized to recognize mRNAs targeted by these 6 DEMis. Next, targeted mRNAs were crosschecked against the DEMs identified previously, and 18 DEMs were recognized. Finally, we established a ceRNA regulatory network containing 8 DECs, 6 DEMis, and 18 DEMs in TNBC using Cytoscape 3.7.1 (Fig. 4).

**Fig. 5:**
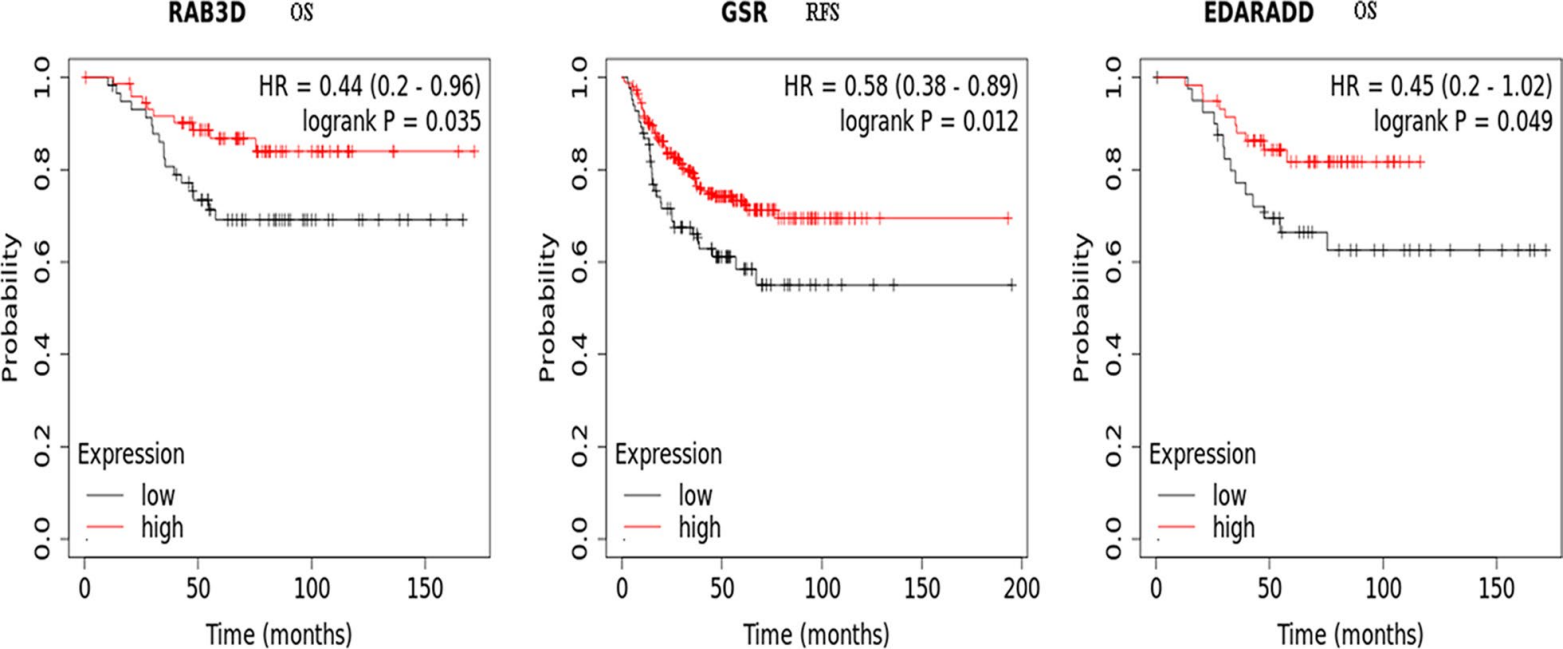
Kaplan‒Meier curves of the survival-related differentially expressed mRNAs in the ceRNA network. The OS of the RAB3D gene (**A**); the RFS of the GSR gene (**B**); the OS of the EDARADD gene (**C**)

The next step was to perform survival analysis of related DEMs. To investigate the expression of the DEMs and their prognostic value in BC, we retrieved mRNA expression profile data and corresponding overall survival (OS) and recurrence-free survival (RFS) data of clinical samples from the Kaplan‒Meier Plotter database. Then, a survival analysis was performed for the 18 DEMs from the ceRNA network. RAB3D and EDARADD were significantly correlated with OS, and GSR was significantly correlated with RFS in patients with BC (Fig. 5). High expression levels of RAB3D and EDARADD predicted better OS. Meanwhile, high expression levels of GSR predicted better RFS. Therefore, these three mRNAs and their corresponding genes were the most likely to be target genes regulated by DECs through the ceRNA network in TNBC.

**Fig. 6:**
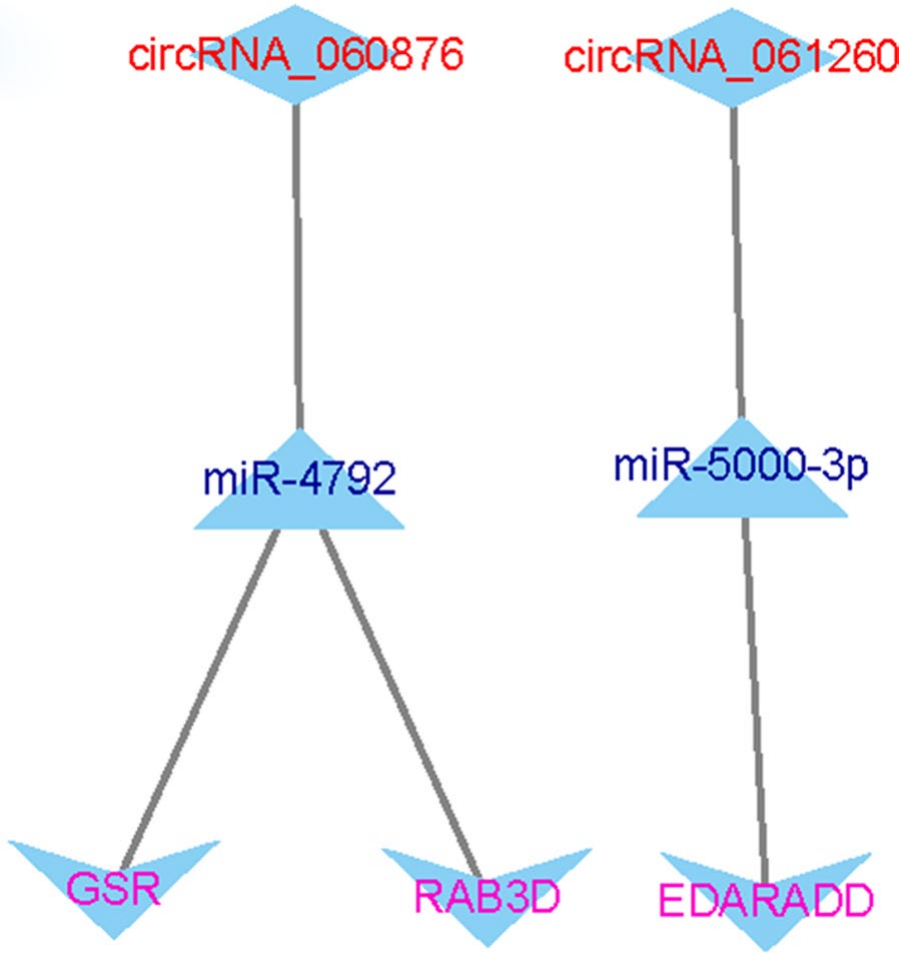
Survival-associated circRNA-miRNA‒mRNA regulatory network in TNBC. The network consists of two DECs (hsa_circRNA_061260 and hsa_circRNA_060876), two DEMis (hsa-miR-5000-3p and hsa-miR-4792), and three mRNAs (GSR, RAB3D, and EDARADD)

Based on the results of survival-related DEMs, we identified the corresponding genes that were also associated with survival outcomes. The three identified genes (RAB3D, EDARADD, and GSR) may be important biomarkers in the prognosis of TNBC. Then, we constructed a survival-related ceRNA network based on these th–ree genes. As shown in Fig. 6, three ceRNA regulatory axes containing two DECs (hsa_circRNA_061260 and hsa_circRNA_060876), two DEMis (hsa-miR-5000-3p and hsa-miR-4792), and three mRNAs (GSR, RAB3D, and EDARADD) were identified. These regulatory modules from the survival-related ceRNA network included the hsa_circRNA_060876/hsa-miR-4792/GSR regulatory axis, hsa_circRNA_060876/hsa-miR-4792/RAB3D regulatory axis, and hsa_circRNA_061260/hsa-miR-5000-3p/EDARADD regulatory axis.

**Fig. 7:**
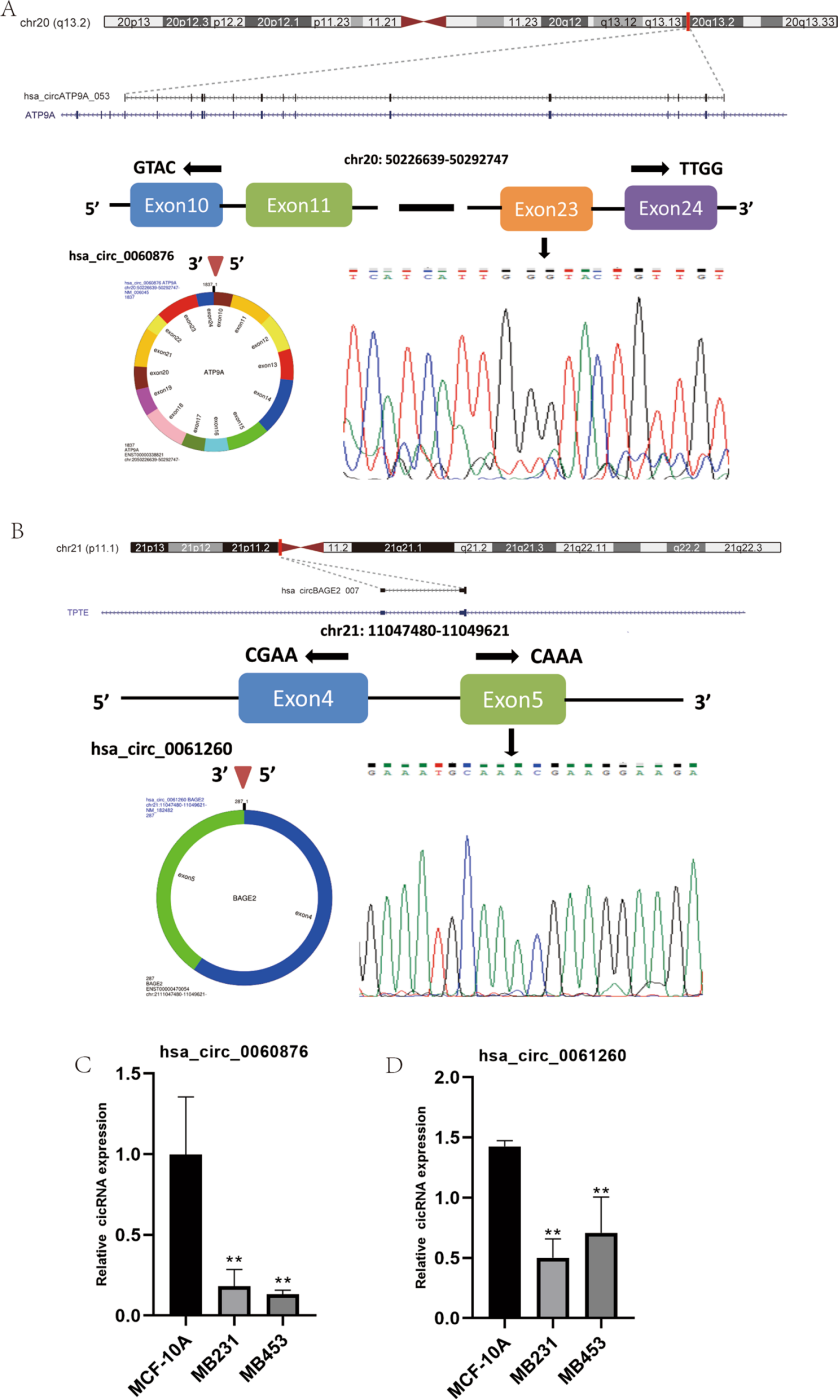
Detailed information on the circRNAs and their back-splice junction sites. The location and corresponding splicing junction site of hsa_circ_0060876 (A) and hsa_circ_0061260 (**B**). The expression levels of hsa_circ_0060876 (**C**) and hsa_circ_0061260 (**D**) in MCF-10 A, MDA-MB-231 and MDA-MB-453 cell lines by RT‒PCR. **P ≤ 0.01

Finally, to validate the results of candidate DECs potentially dysregulated in TNBC cells, we first performed RT‒qPCR in the following cell lines: MCF-10 A, MDA-MB-231, and MDA-MB-453. We selected two DECs for further analysis and conducted Sanger sequencing to confirm the unique back-splicing junction sites of these two DECs (Fig. 7A–B). The DEC hsa_circRNA_061260 is located on chr21: 11,047,480–11,049,621, composed of exon 4 and exon 5. The DEC hsa_circRNA_060876 is located on chr20: 50,226,639–50,292,747, composed of exons 10 to 24. The PCR results revealed that the expression levels of both hsa_circRNA_061260 and hsa_circRNA_060876 were downregulated in the MDA-MB-231 and MDA-MB-453 cell lines compared to the MCF-10 A cell line (p < 0.01; Fig. 7C–D).

## Discussion

ALN metastasis in TNBC predicts poor prognosis. Understanding gene expression regulation may contribute to a promising new direction in therapy. However, the underlying metastatic mechanism is not well defined. In recent years, WTS has become one of the most practical and effective techniques that has enabled researchers to identify driver genes in several types of malignant tumors [14–17]. Previous studies have demonstrated that circRNAs may play vital roles in gene expression and signaling pathways and are involved in the activation and progression of tumors [18]. However, the role of circRNAs in the ALN metastasis of TNBC remains unknown. Herein, we profiled mRNAs, miRNAs, and circRNAs in tumor samples of both nonmetastatic ALN and metastatic ALN patients and focused on circRNAs that are associated with the development of ALN metastasis in TNBC.

In our study, we first employed comprehensive WTS to obtain differentially expressed mRNA, miRNA, and circRNA expression data for paired ALN-positive and ALN-negative TNBC tumor tissues. As a result, we identified several DEMs, DEMis, and DECs in ALN-positive compared with ALN-negative TNBC tumor tissues. Functional enrichment analysis of the DEMs revealed that these mRNAs mainly function in the cell cycle, TNF signaling pathway, breast cancer, p53 signaling pathway, and NOTCH signaling. In cancer cells, cell cycle control is lost, thus allowing continuous replication, and cell cycle control may play a vital role in cancer treatment [19]. CDK4/6 inhibitors act on cell cycle checkpoints, and several preclinical studies have presented their therapeutic capability in TNBC [20]. The Notch signaling pathway also affects cancer cell activation, migration, invasion, metastasis, and resistance to therapy in TNBC. This pathway is also a potential therapeutic target for TNBC [21]. DEMs that are enriched in these pathways might also be associated with the carcinogenesis and tumor progression of TNBC, and further investigation of these DEMs may identify therapeutic targets to treat TNBC.

Recently, some research on circRNA-related ceRNA networks in breast cancer has been reported [7, 9, 22–24]. However, the involvement of this kind of ceRNA network in ALN metastasis in TNBC remains unclear. Based on the candidate top-ranked DECs and the bioinformatic predictions of the interactions between mRNAs, miRNAs, and circRNAs, a ceRNA network was constructed. We found that three DEMs (RAB3D, EDARADD, and GSR) contained in the ceRNA network were significantly associated with OS or RFS in TNBC. Then, a survival-related ceRNA regulatory network was established. As shown in the survival-related ceRNA network, hsa_circRNA_060876 might function as a ceRNA of two DEMs (GSR and RAB3D) when it is bound to hsa-miR-4792, and hsa_circRNA_061260 might function as a ceRNA of one DEM (EDARADD) when it is bound to hsa-miR-5000-3p. Although research on the roles that the above two DECs play in cancer is limited, their targeted DEMis and DEMs have been shown to have important roles in many cancers. For example, GSR was found to act as an oncogene in many cancers, such as cervical cancer, hepatocellular carcinoma, and colorectal cancer [25–27]. Evidence has shown that the expression of RAB3D is upregulated by the ceRNA mechanism in non-small cell lung cancer, osteosarcoma, renal cell carcinoma, and melanoma, leading to tumor progression [28–32]. RAB3D was reported as a hub gene that is involved in many ceRNA networks. It may also play an important role in the ALN metastasis of TNBC. Meng Li et al. found that EDARADD expression is involved in the progression of tongue squamous cell carcinoma [33]. Future mechanistic studies are needed to clarify the role of these DEMs in the ALN metastasis of TNBC.

This study has some limitations. Although candidate DECs were found to be strongly related to ALN metastasis in TNBC, further in vitro and in vivo experimental validation is required to explore the underlying biological mechanisms of the DECs, DEMis, and DEMs. Additionally, our data were extracted from WTS profiles based on three paired tumor samples, and the results were obtained by bioinformatic analysis. Large-scale clinical data are needed for further validation.

In conclusion, our study investigated the molecular mechanism of ALN metastasis in TNBC using WTS. A survival-related ceRNA network was established, and two novel DECs were identified as possible prognostic predictors and potential therapeutic targets of ALN metastasis in TNBC. These findings provide novel ideas for clarifying the mechanisms, achieving early diagnosis and identifying therapeutic targets of ALN metastasis in TNBC

## Data Availability

All data were obtained from whole-transcriptome sequencing.

## Abbreviations

ALN: axillary lymph node
TNBC: triple-negative breast cancer
WTS: whole transcriptome sequencing
DEMs: differentially expressed messenger RNAs
DEMis: differentially expressed microRNAs
DECs: differentially expressed circular RNAs
ceRNA: competing endogenous RNA
KEGG: Kyoto Encyclopedia of Genes and Genomes
OS: overall survival
RFS: recurrence-free survival
PCR: polymerase chain reaction
