# Correction: Lymphatic metastasis-associated circRNA‒miRNA‒mRNA network for exploring the pathogenesis and therapeutic target of triple negative breast cancer based on whole-transcriptome sequencing analysis: an experimental verification study

**DOI:** 10.1186/s12967-023-04025-6

**Published:** 2023-03-07

**Authors:** Jiayue Luo, Dong Cao, Chuwen Hu, Zhen Liang, Yuanping Zhang, Jianguo Lai

**Affiliations:** 1 Guangdong Provincial People’s Hospital, Guangdong Academy of Medical Sciences, 106 Zhongshan Er Road, Yuexiu District, Guangzhou, 510080 Guangdong China; 2 Department of Breast Surgery, Guangzhou Women and Children’s Medical Center, Guangzhou Medical University, Guangzhou, 510623 Guangdong China; 3 Department of Anesthesiology, Sun Yat-Sen Memorial Hospital, Sun Yat-Sen University, Guangzhou, 510120 Guangdong China

**Correction: Journal of Translational Medicine (2022) 20:508** https://doi.org/10.1186/s12967-022-03728-6

Following publication of the original article [1], we have been notified that affiliation of the first author should be modified. They should be as follows:

Jiayue Luo^2†^, Dong Cao^3†^, Chuwen Hu^3^, Zhen Liang^2^, Yuanping Zhang^2^ and Jianguo Lai^1*^

Jiayue Luo

Department of Breast Surgery, Guangzhou Women and Children’s Medical Center, Guangzhou Medical University, Guangzhou, 510623, Guangdong, China

Dong Cao

Department of Anesthesiology, Sun Yat-Sen Memorial Hospital, Sun Yat-Sen University, Guangzhou, 510120, Guangdong, China

Chuwen Hu

Department of Anesthesiology, Sun Yat-Sen Memorial Hospital, Sun Yat-Sen University, Guangzhou, 510120, Guangdong, China

Zhen Liang

Department of Breast Surgery, Guangzhou Women and Children’s Medical Center, Guangzhou Medical University, Guangzhou, 510623, Guangdong, China

Yuanping Zhang

Department of Breast Surgery, Guangzhou Women and Children’s Medical Center, Guangzhou Medical University, Guangzhou, 510623, Guangdong, China

Jianguo Lai

ORCID: http://orcid.org/0000-0002-0120-0245 laijianguo@gdph.org.cn

Guangdong Provincial People’s Hospital, Guangdong Academy of Medical Sciences, 106 Zhongshan Er Road, Yuexiu District, Guangzhou, 510080, Guangdong, China
